# 

**Published:** 1948

**Authors:** 


Vol. LXV. No. 233. SPRING, 1948.

### The Bristol Medico-Chirurgical Journal

PUBLISHED UNDER THE AUSPICES OF

THE BRISTOL MEDICO-CHIRURGICAL SOCIETY.

*Editor:* J. A. NIXON, *C.M.G.*, M.D., F.R.C.P.

WITH WHOM ARE ASSOCIATED

A. RENDLE SHORT, M.D., B.S., B.Sc., F.R.C.S.

W. MELVILLE CAPPER, M.B., B.S., F.R.C.S.

FREDERICK SUTTON, *M.C.*, M.D., F.R.C.P.

E. WATSON-WILLIAMS, *M.C.*, Ch.M., F.R.C.S., *Editorial Secretary.*

BRISTOL:

J. W. ARROWSMITH LTD., QUAY STREET & SMALL STREET

Price Four Shillings net.

